# Influence of Motor Planning on Distance Perception within the Peripersonal Space

**DOI:** 10.1371/journal.pone.0034880

**Published:** 2012-04-24

**Authors:** Wladimir Kirsch, Oliver Herbort, Martin V. Butz, Wilfried Kunde

**Affiliations:** 1 Department of Psychology, University of Würzburg, Würzburg, Germany; 2 Cognitive Modeling, Department of Computer Science, University of Tübingen, Tübingen, Germany; Queen Mary University of London, United Kingdom

## Abstract

We examined whether movement costs as defined by movement magnitude have an impact on distance perception in near space. In Experiment 1, participants were given a numerical cue regarding the amplitude of a hand movement to be carried out. Before the movement execution, the length of a visual distance had to be judged. These visual distances were judged to be larger, the larger the amplitude of the concurrently prepared hand movement was. In Experiment 2, in which numerical cues were merely memorized without concurrent movement planning, this general increase of distance with cue size was not observed. The results of these experiments indicate that visual perception of near space is specifically affected by the costs of planned hand movements.

## Introduction

Visual perception of spatial attributes is traditionally assumed to be determined by optical and ocular-motor information. However, increasing evidence suggests that in addition to variables relating to the visual system, the way we intend and are able to act in a particular situation may affect the way we perceive the environment in that situation. For example, research on the perception of extrapersonal space (henceforth also called far space) showed that hills appear steeper and egocentric distances further if people are encumbered by wearing a heavy backpack [1], [2]. In sport, the perception of external spatial characteristics, such as balls or goals, appears to depend on the performance of athletes (e.g., [3]). For example, Witt and colleagues [4] found that performance in golf was positively correlated with the perceived size of the hole. Similar results are also reported in other sports, such as in American football [5] and in softball [6].

Visual perception of space within reach (henceforth also called near or peripersonal space) also appears to vary. Several studies using different paradigms reported plasticity phenomena related to tool use. One line of evidence stems from studies using a crossmodal (e.g., visual-tactile) stimulation paradigm in right-brain damaged patients with extinction (reviewed in, e.g., [7], [8]). These patients are unable to detect a contralesional stimulus when another stimulus is simultaneously presented on the ipsilesional side (called extinction). Farnè and Làdavas [9], e.g., showed that immediately after using a tool (a 38-cm-long rake) visual stimuli presented at the tip of the tool on the ipsilesional side induced a stronger contralesional tactile extinction than before tool use. This result has been interpreted as evidence for the extension of the peripersonal space of the hand along the tool (cf. also [10]). The authors also reported that the extinction was reduced after a resting period, during which the tool had not been used, indicating a backward contraction of the peripersonal space. Comparable findings are also reported in other studies using similar crossmodal stimulation paradigms, which studied clinical (e.g., [11]) as well as normal populations of participants [12], [13].

Changes of visual perception following tool use are also reported by Berti and Frassinetti [14], who examined a neglect patient with right hemispheric damages showing perceptual deficits in near space but not in far space. Using a tool while performing a perceptual judgment led to the extension of neglect from near to far space in this patient. The result has been taken up in subsequent studies, in which healthy participants are asked to bisect lines presented in different distances ranging from near to far space. In peripersonal space, a slight tendency to judge the midpoint of the line to be left of the real midpoint is typically observed (called pseudoneglect, e.g., [15]). This tendency shifts from left to right, with growing distance between line and participant, when a laser pointer is used for judgment [16], [17]. In contrast, when sticks are used for midpoint estimation, no left to right shift with distance was observed [18], [16]. That is, as in near space, participants tend to perceive the midpoint of the line as being left of the real midpoint. This has been interpreted as being indicative of an expansion of near space due to tool use (cf. also [19]).

Another experimental approach has been pursued by Witt and colleagues [20], [21], [22]. In one study [20] (Exp. 2), the task was to estimate a target distance by means of a reproduction method and to then perform a pointing movement towards this target. Movements included either pointing with a finger or pointing with a conductor's baton. Distances were judged to be shorter in the baton condition than in the finger condition (cf. also [22]). Additionally, the authors report that even the participant's imagination or anticipation of using a tool may be sufficient to induce extension of near space [21].

These studies seem to point to the plasticity of visual awareness of spatial attributes which cannot be understood as simply a function of monocular and binocular visual factors, such as accommodation, convergence, or relative size. Instead, it seems to depend on goals, costs, and possibilities of actions planned in the context of a perceptual act (e.g., [3], [8]). Even though this basic assumption appears to be supported by numerous findings, the exact mechanisms of interaction between perception and action are not well understood. Researchers more or less explicitly assume a kind of scaling process, which relates visual information of the perceiver to some relevant aspects of his real or potential action or of his motor apparatus (cf. [3], [8], [16], [19], [22], [24], for similar assumptions from related areas see e.g., [25], [26]). The basic idea of such a scaling of sensory information depending on motor processes is not new and can be found in works of several renowned researchers, such as of Lotze and Helmholtz (see e.g., [23] for a historical review). Recently, Proffitt [3] (see also [22], [24]) suggested that, for example, the same spatial distance can be scaled differently and is thus perceived differently depending on the intention of the agent. If the perceiver is intending to walk, a distance will be scaled according to the energetic costs that are needed to cover this distance. On the other hand, if the perceiver intends to throw a ball or to reach for an object, the distance will be scaled according to throwing effort or reaching ability, respectively.

One potentially important and interesting aspect of this issue has not received much attention in studies thus far – a possible dependence of the structure of near space on planning of motor activity *within* this space *without* a tool extending the effective reach of the perceiver (but see [27] for an exception). Although many studies examined perception of space within reach, the main focus of research was on a possible interaction between near and far space following tool use, as mentioned. Accordingly, the results are often interpreted as being indicative of temporary near space extension, whereas a part of far space is remapped as a part of near space (e.g., [8], [14]). Simultaneously, tools are typically considered to be incorporated into the body schema (e.g., [10]), suggesting that reported effects of tool use on perception may basically be related to changes of planning and/or executing a joint movement, rather than to tools or reachability per se. If so, tool use would be only one of many possible variables, which may affect the perception of near space. For instance, in contrast to a typical expansion phenomenon associated with tool use, one may expect that stimuli presented in peripersonal space can be perceived as being further away under certain conditions (i.e., the subjective representation of reaching space may be compressed). Some indices for such an effect are reported by Lourenco and Longo [19]. Participants were asked to bisect lines presented in varying distances by means of a laser pointer. Wearing of a weight (2.27 kg) on the wrist during the distance judgments was expressed in a more gradual shift of the tendency from left to right with distance (see above) compared with a control condition, whereas at near distances a stronger rightward bias was observed. This result was assumed to represent a compression of near space following increasing effort related to the acting effector. Thus, reaching ability may not be the exclusive variable that affects spatial perception of objects located close to the observer.

Against this background, we aimed to examine the role of motor planning on perception of spatial distances in near space. We assumed that in addition to reachability, other variables, such as energetic costs, may also affect perception of distances, as demonstrated in studies of extrapersonal space.

## Experiment 1

Energetic factors of walking appear to be taken into account in perception of hills and distances in extrapersonal space (reviewed in, e.g., [28] and [3]). The basic finding is that an increase of anticipated metabolic energy is associated with a suppression of the perceived space. That is, hills appear to be steeper and distances further if costs of action increase (e.g., by a heavy backpack and / or by fatigue). However, to our knowledge, the role of action costs in perception of peripersonal space has not been directly investigated. Action costs are assumed to play an important role in planning of hand movements and they can even be more weighted than costs of locomotion [29]. For instance, stereotypical movement characteristics, such as linear position, biphasic acceleration and bell-shaped velocity trajectories, are typically considered to be a result of optimal (i.e., cost-effective) planning and control strategies [30], [31], [32]. Costs usually increase if movement duration and / or amplitude increase, or the more muscles are involved and the more intensively the muscles are strained (e.g., [31]). Based on the findings of effort-related perceptual modulation observed in extrapersonal space, we aimed to test whether planning a movement associated with varying effort may affect the perception of a spatial distance in near space. We asked whether energetic costs, as defined by movement magnitude, cause changes in perception. Some indices from the study of Lourenco and Longo [19] (see above) support this possibility.

In each trial participants initially received a cue which informed them about the amplitude of a movement that had to be executed after a distance judgment. Thus, during distance estimation, participants had to prepare, or at least keep in mind, the amplitude of a movement. The spatial distance between two stimuli was estimated by the alignment of two additional stimuli presented orthogonally to the given distance (cf. [20], [21]). After the distance estimate was made, participants had to perform the movement of instructed amplitude. The critical manipulation was related to the amplitude of the movement, which was either identical to the stimulus distance, or 3, 2, or 1 cm smaller or larger than the stimulus distance. We assumed that a gradual increase in movement amplitude (i.e., an increase in movement costs) would cause a gradual compression of the subjective representation of the near space whereby a given distance should appear further away.

### Methods

#### Ethics Statement

All participants volunteered and provided verbal (Exp. 1) or written (Exp. 2) informed consent. The study was conducted in accordance with German Psychological Society (DGPs) ethical guidelines (2004, CIII). According to these guidelines informed consent can be written as well as verbal. This research was also reviewed and approved by the German Research Council (DFG, project number KI 1620/1-1) which did not require Institutional Review Board approval.

#### Participants

The sample consisted of twenty-four participants (19 female, 5 male). Most were students of the University of Würzburg. The mean age was 24.8 years, ranging from 19 to 36 years of age. Each participant received an hourly payment or course credit for participation.

#### Apparatus

**Figure 1:**
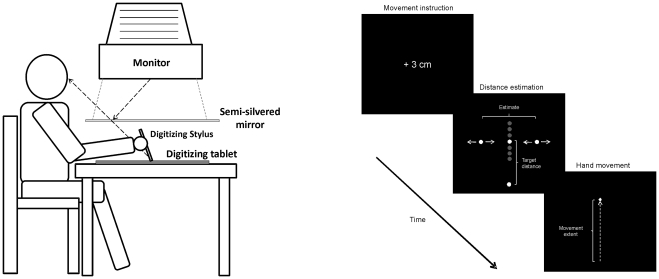
Schematic illustration of the used apparatus (left) and of the trial procedure (right). Note, circles shown in grey are potential target positions, which were not visible in this example. During the hand movement only the virtual position of the stylus was presented (shown here at the end of the movement). The movement instruction in the given example (+3 cm) requires participants to prepare a movement that is 3 cm longer than a movement to the target.

The apparatus consisted of a digitizing tablet (Wacom Intuos 2 A4) and a monitor / mirror system (see Figure 1, left; cf. also [33]). A semi-silvered mirror was positioned midway between the tablet and the screen (about 23 cm above the tablet) so that virtual images of the display appeared in the plane of the tablet. When the light was dimmed, the mirror prevented direct view of the arm. The monitor was set to a resolution of 1024×768 picture elements (*PEL*). One *PEL* measured about 0.38 mm on the screen. The relation between the stimulus position indicating the position of the stylus and the actual position of the stylus was aligned so that feedback corresponded approximately to the actual stylus position (i.e., there was no manipulation of visual feedback).

#### Procedure and design

Participants sat in front of the apparatus so that the position of the body midline corresponded with the middle of the screen. The trial procedure is schematically illustrated in Figure 1 (right). At the beginning of each trial, the participant moved the stylus to the start position, which was located on the tablet next to the body at the level of the body midline. A number was then displayed which informed the participant to hit a target circle which would appear later (“0 cm”), to overshoot the target circle by 1, 2, or 3 cm (“+1 cm”, “+2 cm”, “+3 cm”), or to undershoot the target circle by 1, 2, or 3 cm (“−1 cm”, “−2 cm”, “−3 cm”). Additionally, a short text informed the participant that the experiment will continue as soon as the space bar was pressed.

After the participant pressed the space bar, the start circle and target circle appeared in the middle of the otherwise black screen. Both circles were white and had a diameter of approximately 4 mm. The position of the start circle was always constant and corresponded to the starting position of the stylus. The position of the target circle served as an anchor for stylus movements and varied from trial to trial.

After the participant pressed a button on the stylus, two additional white circles (4 mm) appeared to the left and right of the target circle (i.e., they successively arose from the target circle with each button press). The participants had to adjust the horizontal distance between the left and right circle by pressing stylus buttons so that it corresponded to the vertical distance between start and target circle. A new or continuous pressing of one key caused an increase of the distance between the horizontal circles, whereas the other key could be used to decrease the distance. During this ascending adjustment procedure the positions of the horizontal circles were always symmetrical in respect to the given target (i.e., the right and left circles were always equidistant in respect to the target). The adjustment procedure was completed by pressing a marked key on a keyboard.

Following this key press, all stimuli disappeared and the current stylus position was shown in form of a green circle (4 mm). This change of the display was the signal for the participant to move the stylus according to the movement instruction. After finishing the movement, participants were instructed to press a key on the stylus. Following this key press a red circle, which had a diameter of approximately 2 mm, appeared at the starting position, in response to which participants had to move the stylus back to the start position in order to initiate the next trial.

There were two independent variables. First, the *movement instruction* could be to overshoot the target by 1, 2, or 3 cm, undershoot the target by 1, 2, or 3 cm, or hit the target exactly. Second, the *stimulus distance*, which was the distance between the start circle and the target circle, varied between 252 *PEL* (∼ 9.6 cm) and 414 *PEL* (∼ 15.7 cm) in steps of 27 *PEL* (∼1 cm).

The experiment consisted of 5 blocks of 49 trials, in which each combination of movement instruction and stimulus distance was presented once in randomized order. Additionally, nine practice trials that did not enter the analyses were administered before the start of the experiment. The breaks between blocks of trials were adjusted to individual demands. The experiment lasted about 1 hour (due to the self-paced nature of the procedure there were considerable individual differences).

#### Data Recording and Analysis

In each trial, the amplitude of the stylus movement (*movement amplitude*) was extracted when participants pressed the stylus button after the movement. Additionally, the difference between the distances between the horizontal and the vertical stimuli was recorded after the adjustment of the horizontal stimulus distance (*constant perceptual error*). Positive perceptual errors denote overestimations of the vertical distance, negative perceptual errors denote underestimations of the vertical distance. Trials in which estimated distances or movement amplitudes were smaller than 10 *PEL* (0.38 cm) or larger than 800 *PEL* (30.4 cm) were excluded from analysis. Responses that were more than 3 *SD* above or below a participant's mean with respect to stimulus distance and movement instruction condition were also considered as outliers and were thus discarded from further analyses (0.6%).

## Results

### Movement amplitude

The medians of movement amplitudes and of perceptual errors were computed for each participant and each combination of stimulus distance and movement instruction. In order to ensure that participants followed the movement instruction we initially analyzed the movement amplitudes using an ANOVA with stimulus distance (7 levels) and movement instruction (7 levels) as within-subject factors. As expected, this ANOVA revealed significant main effects of stimulus distance and of movement instruction with *F*(6, 138) = 2310.2, *p*<0.001 and *F*(6, 138) = 289.5, *p*<0.001 respectively. Movement amplitude systematically increased with stimulus distance and with an increase of the amplitude required by the movement instruction (see Table 1 for mean values). Pairwise comparisons (*t*-tests, *df* = 23) further indicated that for both factors all conditions were significantly different from each other (all *p*<0.001).

**Table 1:** Mean movement amplitude according to the target distance and movement instruction conditions. Corresponding standard deviations are shown in brackets.

		Stimulus Distance													
		1 (252 *PEL*)		2 (279 *PEL*)		3 (306 *PEL*)		4 (333 *PEL*)		5 (360 *PEL*)		6 (387 *PEL*)		7 (414 *PEL*)	
MovementInstruction	−3	171	(38)	196	(30)	221	(28)	250	(31)	276	(29)	311	(35)	334	(28)
	−2	194	(19)	221	(21)	248	(21)	278	(21)	303	(19)	329	(22)	358	(21)
	−1	219	(17)	244	(18)	270	(13)	296	(16)	325	(16)	356	(22)	380	(17)
	0	253	(9)	281	(9)	306	(8)	335	(6)	362	(8)	391	(8)	417	(9)
	+1	291	(13)	321	(13)	342	(25)	374	(13)	401	(14)	428	(23)	458	(13)
	+2	318	(21)	346	(21)	373	(22)	395	(23)	420	(24)	453	(22)	482	(18)
	+3	342	(30)	372	(28)	400	(30)	426	(27)	453	(25)	482	(29)	495	(43)

### Constant perceptual error

An analysis of variance (ANOVA) performed on perceptual errors with within-subject factors stimulus distance and movement instruction revealed a significant main effect for the factor stimulus distance, *F*(6, 138) = 14.9, *p*<0.001, and more importantly, a significant effect for the factor movement instruction, *F*(6, 138) = 3.9, *p* = 0.001. Moreover, both factors did not interact, *F*(36, 828) = 0.8, *p* = 0.825. Participants generally tended to overestimate the given stimulus distance and this tendency increased with an increase in stimulus distance. Mean perceptual error values were 53 (*SD* = 38), 55 (*SD* = 41), 59 (*SD* = 46), 62 (*SD* = 47), 71 (*SD* = 53), 73 (*SD* = 56), and 80 (*SD* = 59) *PEL*, for stimulus distances 1 to 7 respectively (for pairwise comparisons see Table S1).

**Figure 2:**
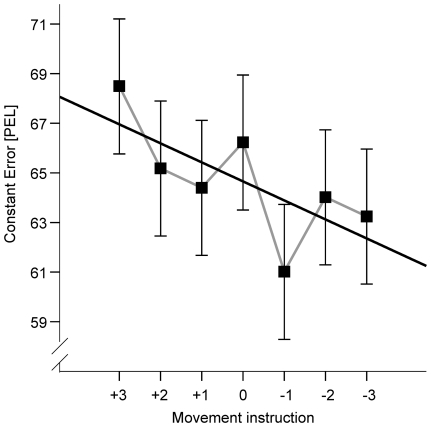
Results of Experiment 1. Mean constant error as a function of the movement instruction. Black line is regression line fitted to the shown means. Error bars reflect within-subjects confidence intervals (according to [34]).

The impact of the movement instruction on perceptual judgments is illustrated in Figure 2. As predicted, the tendency to overestimate the vertical distance was the greater pronounced, the larger the amplitude of the planned movement was. This relationship could be approximated by a linear function as indicated by a significant linear contrast, *F*(1, 23) = 7.4, *p* = 0.012. In particular, if the two extreme and the intermediate conditions (i.e., “−3”, “0” and “+3”) are considered, the expected linear pattern is obtained. The difference between “−3” and “+3” conditions also proved to be significant, *t*(23) = 2.9, *p* = 0.008 (for all other comparisons see Table S2). Nevertheless, systematic deviations from a strong linearity seemed also to be present as indicated by a significant polynomial trend of degree 6, *F*(1, 23) = 4.7, *p* = 0.040. This contrast suggests that systematic judgment errors were made that resulted in overestimations when movement extents of 3, 0, −2, and −3 cm were instructed, and they resulted in underestimations for the remaining movement extents when compared with the linear trend. Thus, when considering movement instruction conditions with different signs separately and ignoring the “0” condition, a trend towards a stronger overshoot with an increase in digit magnitude can be observed. This observation suggests that the deviation from an expected linear relation between the amplitude of the preplanned movement and the perceived visual distance is not due to random noise.

### Discussion

The present experiment revealed that the amplitude of a planned movement affects visual perception of distances. Generally, participants overestimated the vertical distances. This bias likely reflects the horizontal-vertical illusion, which is a general tendency to overestimate the length of vertical compared to horizontal lines of equal size (e.g., [35]). An increase of the overestimation bias with stimulus distance conceivably indicates an increase of this illusion effect. Because these effects, which reflect mostly the impact of optical variables, were independent of motor planning, we do not consider them further.

More importantly, the manipulation of amplitude of movements following perceptual judgments affected distance estimations as predicted: the larger the amplitude of the planned movement, the stronger the tendency of the participants to overestimate a given distance. Thus, the results are in line with the hypothesis that movement planning in general, and anticipated movement costs in particular, can affect the subjective representation of a spatial distance. Moreover, the direction of the effect corresponds well with the assumption of a scaling process according to which effort or action costs may be used as reference units for perception.

In addition to this proposed linear scaling of visual distance with movement amplitude, the results include another nonlinear component that we did not predict. Considering movement instruction conditions including digits with different signs separately, we observed a trend towards a stronger overshoot with an increase in digit magnitude. This trend appeared to hold true only when the “0” condition was ignored. The higher order polynomial captures these trends and suggests that one origin of the observed effect of movement instruction on perceptual judgments may be related to the processing of digits and their signs, which served as movement cues in Experiment 1. For instance, the pure magnitude of the digit may act as an anchor which triggers an increase in perceived distance with an increase in digit magnitude. That is, the observed nonlinear trend may be due to an impact of the digit magnitude on perceptual judgments.

## Experiment 2

To evaluate whether the instructional cue including a digit and a sign affected distance estimations systematically and independent of planning a target-related movement, we performed a control experiment. Participants performed the same task as in Experiment 1 with one exception. Instead of planning and executing a hand movement related to the target, they had to memorize the combination of the digit and its sign, which served as movement instruction in Experiment 1, and to reproduce it after the distance was estimated. We aimed to discriminate between three hypotheses. (1) The perceptual bias resulting from the movement instruction in Experiment 1 may be a result of a compound influence of two factors: the processes associated with movement planning and the memory processes involved in the maintenance of the movement cue. If so, the results of Experiment 2 should reveal a systematic influence of the instructional cue on distance estimation that, however, should differ from the effect found in Experiment 1. In particular, a linear trend towards an increase of estimate magnitude from “–3” to “+3” condition (i.e., with a former increase with movement amplitude) should no longer be observed. (2) However, it is also possible that the effect observed in Experiment 1 can be fully explained by an influence of the cue alone. In this case, a systematic effect of cue should be observed to be as similarly pronounced in Experiment 2 as in Experiment 1. (3) Moreover, it is also possible that the cue did not affect distance judgments at all, so that no systematic effect of the cue is predicted in Experiment 2.

### Methods

#### Participants

Twenty-four participants were recruited (18 female, 6 male). One of these participants also participated in Experiment 1. We also performed all analyses reported below excluding this subject. In doing so we did not observe any changes in the main pattern of results. That is, all significant results were still significant, whereas all non-significant results remained non-significant. The mean age of the participants was 25.6 years (range: 19 to 43). They received an hourly payment for participation.

#### Procedure and design

Experiment 2 was identical to Experiment 1 with the exception that participants had neither to plan nor to execute the stylus movement in Experiment 2. Instead, the instruction required them to memorize the number, which served as movement instruction in Experiment 1, and to write it down on a sheet of paper after the distance judgment. That is, as in Exp. 1, participants saw a signed digit and estimated distances initially. Following perceptual judgment, however, they were asked to reproduce the digit and the respective sign instead of executing a stylus movement: a short instruction was presented on the display informing the participant that the memorized item had to be written down. This text also required the participant to press a key on a keyboard in order to complete the reproduction procedure. Following this key press a red circle (2 mm) appeared at the starting position. In response to this stimulus participants had to move the stylus to the start position in order to initiate the next trial. The duration of Exp. 2 was comparable to the duration of Exp. 1 (i.e., about 1 hour).

Trials in which the movement instruction cue was incorrectly reproduced were excluded. Moreover, as in Experiment 1, trials with distance judgments of less than 10 *PEL* or of more than 800 *PEL* were discarded. Responses that were more than 3 *SD* above or below a participant's mean with respect to stimulus distance and instruction condition were also considered as outliers and were discarded from further analyses. Altogether 2.5% of the trials were discarded.

## Results

Medians of the perceptual errors were computed separately for each participant, each stimulus distance, and each memorized number condition.

**Figure 3:**
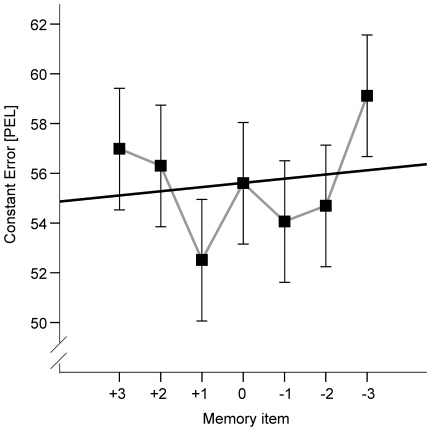
Results of Experiment 2. Mean constant error as a function of the memory item. Black line is regression line fitted to the shown means. Error bars reflect within-subjects confidence intervals (according to [34]).

An ANOVA performed on perceptual error values revealed results which were at first glance similar to those observed in Experiment 1. A main effect of stimulus distance and a main effect of memorized number were significant with *F*(6, 138) = 17.7, *p* <0.001 and *F*(6, 138) = 3.6, *p* = 0.002 respectively. There was no significant interaction between the two factors, *F*(36, 828) = 0.7, *p*  = 0.870. Participants overestimated the vertical distance and this bias increased with an increase in target distance: 43 (*SD*  = 39), 47 (*SD* = 43), 52 (*SD* = 46), 53 (*SD* = 46), 59 (*SD*  =  47), 66 (*SD*  =  49) and 69 (*SD*  =  49) *PEL* for stimulus distances 1 to 7 respectively (for post-hoc tests see Table S3). Although the memorized number significantly affected the distance judgments, the effect of number on distance judgments differed between Experiment 1 and 2. Figure 3 shows the respective means (results of pairwise comparisons can be found in Table S4). An approximately linear decrease of the constant error from “+3” to “−3” conditions observed in Experiment 1 cannot be obtained. This is also substantiated by a non-significant linear contrast, *F*(1, 23) = 0.5 *p* = 0.469. However, a higher order function, including a trend of both extreme and the intermediate conditions (i.e., of “−3”, “+3” and “0”) towards higher values, seems to be present in the given data set and seems to be substantiated by a significant quadratic contrast, *F*(1, 23) = 18.4, *p*<0.001. It is also worth mentioning that a polynomial trend of degree 6 approximated the significance threshold, *F*(1, 23) = 3.5, *p* = 0.073. Thus, apart from the linear trend towards an increase of overestimation from “−3” to “+3” conditions observed in Exp. 1 but not in Exp. 2, both data sets appeared to include a non-linear component that is similarly pronounced in both experiments.

### Joint analysis

In order to test whether the results of both experiments significantly differ in respect to the perceptual judgments, we performed two additional analyses including experiment as a between-subjects factor.

### Interaction of experiment and cue indentity

The first analysis aimed to test the assumption that there may be two variables contributing to the results of the first experiment. The first factor was assumed to be related to the amplitude of the planned movement and should only be present in Experiment 1. The second factor was assumed to be associated with (non-motor) processing and memorizing of the cue itself. As processes associated with the maintenance of the cue during the distance estimation may be expected in both experiments, the “pure” influence of movement planning may be assumed to be captured by differences between both experiments, which should follow a linear increase with an increase in movement amplitude (cf. e.g., [36]). An ANOVA with within-subject factors cue identity and stimulus distance, and the between subject factor experiment, revealed a significant interaction between the factors cue and experiment, *F*(6, 276) = 2.8, *p* = 0.012. The linear contrast for this interaction was also significant, *F*(1, 46) = 6.6, *p* = 0.013. Thus, besides the identity of the cue itself, the amplitude of the planned hand movement also affected distance estimations in Experiment 1 in a predicted way.

### Effect of effort on perceived distance

With a second analysis we aimed to examine how the distance judgments changed over time in both experiments. According to our action-orientated approach, visual perception of near space might be scaled with respect to motor constraints, such as the effort required to execute a movement. Because hand movements had to be performed in Experiment 1 but not in Experiment 2, muscle fatigue, and thus the effort associated to hand movements, should increase more strongly during the course of Experiment 1 than during the course of Experiment 2. Thus, if movement effort affects perceived distance, it can be expected that distance estimates increase during Experiment 1 but not during Experiment 2.

**Figure 4:**
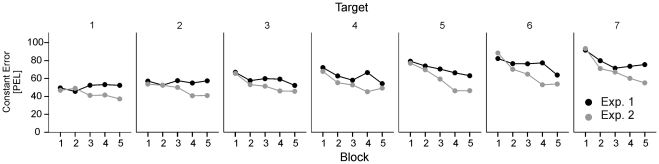
Mean constant error as a function of block of trials in Experiment 1 and Experiment 2.

In line with this assumption, the distance judgments were similarly pronounced in both experiments in the initial blocks of trials, but they diverged over the successive blocks. Although this block x experiment interaction did not reach the significance threshold in the statistical analysis (ANOVA with block, stimulus distance and experiment as factors), *F*(4, 184) = 1.6, *p* = 188, a block x experiment x target distance interaction was significant, *F*(24, 1104) = 1.7, *p* = 0.015. As shown in Figure 4, the relative increase in the magnitude of perceived distance with time in Experiment 1 as compared with Experiment 2 was dependent on stimulus distance to some extent.

### Discussion

By asking participants to keep a signed digit in mind during estimation of a spatial distance, we aimed to test whether digit magnitude and / or digit sign may have an impact on distance judgments and thus, may explain the pattern of results observed in Experiment 1. In fact, we found a significant influence of the cue on distance estimates, which, however, was distinct from the effect of movement cue observed in Experiment 1. In particular, an approximately linear increase in estimated distance from “−3” to “+3” condition, which corresponded to an increase of movement amplitude in Experiment 1, was not observed. In contrast, a nonlinear component, which was associated with a trend to underestimate distances if the cue included small digits except for zero, was present in Experiment 2 as well as in Experiment 1 as indicated by significant polynomial contrasts of degrees two and six. Polynomials of degrees two (quadratic function) and six are similar, but may differ in the number of possible direction changes of the polynomial curves. The highest number of such changes for a quadratic function is one, whereas a polynomial of degree six may have up to five turnings. In Experiment 2, the distribution of mean perceptual error values across the seven cue conditions appears to be best described by a quadratic function, which ignores the central “bump” of the “0” condition (which, however, appeared to be captured by the marginally significant higher order polynomial). In contrast, in Experiment 1 the non-linear trend is superimposed by a linear trend, which seems to facilitate a higher order polynomial and to obstruct a quadratic trend. Thus, increasing trends in judgment errors with an increase in magnitude of digits (expect for zero) were present in both experiments. Accordingly, the magnitude of the memorized digit also affected perception. Thus, the results of Experiment 2 are in line with the hypothesis that both the amplitude of the preplanned movement and the instructional cue had an impact on the perception of spatial distance in Experiment 1.

Moreover, in comparing the results of both experiments we observed that distance judgments differently changed in the course of the experiment. Participants of Experiment 2 successively underestimated a given distance, as compared to participants of Experiment 1. This trend was somewhat differently pronounced at different distances and appeared to fit well into the other results. Assuming that visual information is scaled according to the effort of intended action, one may expect a relative increase in overestimation in Experiment 1 as compared to Experiment 2 due to an increase in muscle fatigue following the increasing number of movements performed in Experiment 1. In other words, a perceived spatial distance successively appeared prolonged because progressively more effort was needed to execute one and the same movement.

The influence of digits on distance estimations reminds of a phenomenon called the *spatial-numerical association of response code* (SNARC) *effect* [37]: in parity (i.e., odd-even) judgment tasks participants typically respond faster with the left hand (or with other effectors operating on the left side of space) than with the right hand (or with other effectors operating on the right side of space) to relatively small numbers, whereas responses to relatively large numbers are typically faster with the right than with the left hand. Such an association between number magnitude and spatial location of response has been observed in a variety of experimental conditions and is usually explained by a spatial correspondence between the position of a number on a left-to-right oriented *mental number line* and the position of response (see e.g., [38] for a review). Our present results appear to resemble reports of a *vertical* version of the SNARC effect – faster responses to the bottom (top) response location when a small (large) number was shown – suggesting modifications of the mental number line metaphor [39], [40]. The tendency towards an overestimation of distance with an increase in number magnitude observed in the present study may thus reflect an interaction between spatial aspects of a representation of numerical magnitude and visual distance perception. For instance, relatively small numbers may be internally represented as “bottom” and relatively large numbers as “top” within *a mental number map* (cf. e.g., [39]). Alternatively, the interaction may also occur on a more abstract conceptual level of magnitude: small numbers may be more associated with close spatial distances, whereas large numbers may be more related to long spatial distances. Although the locus of this interaction is of course not clear, the given paradigm seems to provide a promising approach to investigate the relation between number magnitude representation and spatial perception in a rather direct fashion.

## Discussion

The main purpose of the present experiments was to examine a possible dependence of visual perception of reachable distances on motor planning processes. Based on findings indicating plasticity of extrapersonal space following changes in anticipated effort of action, we tested whether a similar phenomenon is also observable in near space, in which objects are potentially reachable without locomotion. We hypothesized that an increase in movement costs will cause a compression of the subjective space and will cause a given distance to appear prolonged.

The results of Experiment 1 corresponded well with this hypothesis. We observed that the larger the amplitude of a preplanned movement was, the greater the participants tended to overestimate a given target distance. Because the amplitude of the preplanned movement was assumed to reflect anticipated effort, such an assimilation effect suggests that visual information associated with a given target distance was influenced by motor planning processes, whereby it might have been scaled by energetic costs (but see below). However, additional observations raised some doubt about this interpretation. The relationship between the distance estimations and the preplanned movement amplitude seemed to systematically deviate from a predicted linear function. This led us to assume that distance estimations may have been additionally or exclusively affected by the identity of the movement cue. In order to evaluate this issue we performed a control experiment, in which no distance-related hand movements were performed but the instructional cue had to be memorized.

The results of Experiment 2 appeared to substantiate the assumption that in addition to the impact of motor planning, the memory processes associated with the maintenance of a signed digit may also affect distance perception. The cue identity significantly affected distance estimations. Moreover, this effect was similar to the nonlinear trend obtained in Experiment 1. Except for the zero condition, participants showed a tendency towards a decrease of estimate with a decrease in digit magnitude. However, in contrast to the results of Experiment 1, this effect was rather symmetrical in that it appeared to be independent of the cue sign (i.e., of the former movement instruction). Thus, the results suggest that in Experiment 1 the influence of motor planning on distance perception was superimposed by the influence of the magnitude of digit.

The impact of anticipated motor variables on distance perception is also supported by an additional analysis, in which both experiments were compared with respect to changes of perception in the course of the experiment. Participants of Experiment 1, in which hand movements were performed, tended to successively overestimate a given visual distance as compared with participants of Experiment 2. This effect may indicate that an increase in muscular fatigue resulted in an increase of effort needed to achieve a given target. Assuming that perception of near space is scaled depending on effort, the observed increase in perceived distance can be predicted.

These conclusions should, of course, be considered with caution. There are at least three caveats, which may limit the validity and generalization of the results. (1) Because participants were using a motor response (button press) to make perceptual estimates, it cannot be ruled out that the effect of movement instruction on judgments may be limited to perceptual judgments that involve a kind of action as well. In particular, it is possible that the manipulation of motor planning affected the action of judgment rather than perception. Although a “low-level” response-response effect appears to be rather implausible due to different kinds of action (finger movements vs. hand movements), a reciprocal relation between both actions might exist on a more abstract level. For instance, planning a hand movement of relatively large amplitude may promote a button response of a relatively long duration. Due to the use of a type of ascending adjustment procedure, this may theoretically cause a judgment bias such as one found in the present study. This, however, appears to be rather unlikely because participants were able to correct their estimates (e.g., to decrease the horizontal distance) at any time during the adjustment procedure.

(2) We used movement amplitude as a measure of movement costs. Although seemingly plausible, the possibility cannot be excluded that other variables also contributed to the results. For instance, variation of movement amplitude is typically associated with a variation of movement time. Thus, the observed effect of the amplitude manipulation on distance perception might also be caused by factors related to movement time rather than to movement amplitude. Moreover, planning a movement is usually assumed to include an explicit representation of a movement goal (e.g., [41], [42]). Accordingly, because different amplitudes are associated with different movement goals, the mentioned effect might also be spatial in nature, e.g., caused by an assimilation of the distance estimate to the spatial end position of the intended movement. In other words, although the observed effect might be related to the amplitude of a movement, it might include a more abstract level of processing than analyses of movement costs. The observed side effect of numerical magnitude on distance estimates appears to support this possibility.

(3) Another possible caveat is related to eye movements. We did not measure ocular activity and thus, cannot assess its impact on the results. It is known that in simple point-to-point hand movements, eye movements typically precede arm movements (cf. e.g., [43]). Thus, an effect of anticipated hand movement, such as one found in the present study, may be related to intended eye movements rather than to arm movements.

To conclude, in the present study we found indicators that visual perception of spatial distances presented in reachable space is affected by processes associated with planning a hand movement. This result extends previous research demonstrating action-specific plasticity of visual perception to near space. In line with evidence from studies on extrapersonal space, our results suggest that distance perception might be mediated by units of anticipated movement effort. However, further research is needed to describe the impact of motor and non-motor variables on perception of near space in more detail.

## Supporting Information

Table S1 **Post-hoc analyses of constant perceptual errors in Exp. 1.** Results of pairwise comparisons (*t*-tests for dependent measures, *df* = 23) according to the main effect “stimulus distance”. *P*-values are shown.
(DOCX)

Table S2 **Post-hoc analyses of constant perceptual errors in Exp. 1.** Results of pairwise comparisons (*t*-tests for dependent measures, *df* = 23) according to the main effect “movement instruction”. *P*-values are shown.
(DOCX)

Table S3 **Post-hoc analyses of constant perceptual errors in Exp. 2.** Results of pairwise comparisons (*t*-tests for dependent measures, *df* = 23) according to the main effect “stimulus distance”. *P*-values are shown.
(DOCX)

Table S4 **Post-hoc analyses of constant perceptual errors in Exp. 2**. Results of pairwise comparisons (*t*-tests for dependent measures, *df* = 23) according to the main effect “movement instruction”. *P*-values are shown.
(DOCX)
