# Supplementary material for: Influence of Motor Planning on Distance Perception within the Peripersonal Space
Source: PLoS One. 2012 Apr 24;7(4):e34880. doi: 10.1371/journal.pone.0034880 (PMC3335827; doi:10.1371/journal.pone.0034880)
Supplement: Table S2 — Post-hoc analyses of constant perceptual errors in Exp. 1. Results of pairwise comparisons (t-tests for dependent measures, df = 23) according to the main effect “movement instruction”. P-values are shown. (DOCX) [file pone.0034880.s002.docx]

|  | | | Movement Instruction | | | | | | |
| --- | --- | --- | --- | --- | --- | --- | --- | --- | --- |
|  |  |  | -3 | -2 | -1 | 0 | +1 | +2 | +3 |
| Movement  Instruction | -3 |  | - | .468 | .057 | .065 | .470 | .196 | .008 |
|  | -2 |  | - | - | .043 | .111 | .808 | .365 | .025 |
|  | -1 |  | - | - | - | .012 | .087 | .027 | .002 |
|  | 0 |  | - | - | - | - | .333 | .576 | .362 |
|  | +1 |  | - | - | - | - | - | .639 | .070 |
|  | +2 |  | - | - | - | - | - | - | .013 |
|  | +3 |  | - | - | - | - | - | - | - |
