# Supplementary material for: Influence of Motor Planning on Distance Perception within the Peripersonal Space
Source: PLoS One. 2012 Apr 24;7(4):e34880. doi: 10.1371/journal.pone.0034880 (PMC3335827; doi:10.1371/journal.pone.0034880)
Supplement: Table S3 — Post-hoc analyses of constant perceptual errors in Exp. 2. Results of pairwise comparisons (t-tests for dependent measures, df = 23) according to the main effect “stimulus distance”. P-values are shown. (DOCX) [file pone.0034880.s003.docx]

|  | | | Stimulus Distance | | | | | | |
| --- | --- | --- | --- | --- | --- | --- | --- | --- | --- |
|  |  |  | 1 (252 *PEL*) | 2 (279 *PEL*) | 3 (306 *PEL*) | 4 (333 *PEL*) | 5 (360 *PEL*) | 6 (387 *PEL*) | 7 (414 *PEL*) |
| Stimulus  Distance | 1 (252 *PEL*) |  | - | .077 | .001 | .006 | .001 | <.001 | <.001 |
|  | 2 (279 *PEL*) |  | - | - | .002 | .025 | .002 | <.001 | <.001 |
|  | 3 (306 *PEL*) |  | - | - | - | .680 | .025 | <.001 | <.001 |
|  | 4 (333 *PEL*) |  | - | - | - | - | .005 | <.001 | <.001 |
|  | 5 (360 *PEL*) |  | - | - | - | - | - | <.001 | .001 |
|  | 6 (387 *PEL*) |  | - | - | - | - | - | - | .142 |
|  | 7 (414 *PEL*) |  | - | - | - | - | - | - | - |
