# Supplementary material for: Influence of Motor Planning on Distance Perception within the Peripersonal Space
Source: PLoS One. 2012 Apr 24;7(4):e34880. doi: 10.1371/journal.pone.0034880 (PMC3335827; doi:10.1371/journal.pone.0034880)
Supplement: Table S4 — Post-hoc analyses of constant perceptual errors in Exp. 2. Results of pairwise comparisons (t-tests for dependent measures, df = 23) according to the main effect “movement instruction”. P-values are shown. (DOCX) [file pone.0034880.s004.docx]

|  | | | Movement Instruction | | | | | | |
| --- | --- | --- | --- | --- | --- | --- | --- | --- | --- |
|  |  |  | -3 | -2 | -1 | 0 | +1 | +2 | +3 |
| Movement  Instruction | -3 |  | - | .017 | .003 | .036 | .001 | .069 | .178 |
|  | -2 |  | - | - | .716 | .625 | .187 | .412 | .163 |
|  | -1 |  | - | - | - | .384 | .305 | .215 | .061 |
|  | 0 |  | - | - | - | - | .029 | .671 | .301 |
|  | +1 |  | - | - | - | - | - | .037 | <.001 |
|  | +2 |  | - | - | - | - | - | - | .647 |
|  | +3 |  | - | - | - | - | - | - | - |
